# Rapidly Progressive Primary Ovarian Malignant Perivascular Epithelioid Cell Tumor

**DOI:** 10.7759/cureus.101960

**Published:** 2026-01-21

**Authors:** Kenta Sonehara, Takashi Suzuki

**Affiliations:** 1 Obstetrics and Gynecology, Saku Central Hospital Advanced Care Center, Saku, JPN

**Keywords:** case report, malignant pecoma, mtor inhibitor, ovary, perivascular epithelioid cell tumor

## Abstract

Perivascular epithelioid cell tumor (PEComa) is a rare mesenchymal neoplasm characterized by a unique dual differentiation toward smooth muscle and melanocytic lineages. While PEComas can arise in various gynecologic organs, primary malignant ovarian PEComa is exceptionally rare and shows a wide spectrum of clinical behaviors. We report a case of a rapidly progressive primary ovarian malignant PEComa with an extremely poor prognosis. A 73-year-old woman presented with fever and malaise. A CT scan incidentally revealed a left ovarian tumor, for which she was referred to our department. Tumor markers were within normal limits, but MRI and CT suggested a left ovarian malignancy. A total hysterectomy and bilateral salpingo-oophorectomy were performed. Pathological examination revealed a 9 cm solid mass with necrosis, cytologic atypia, high mitotic activity (14/HPF), and vascular invasion. Immunohistochemistry was positive for both melanocytic markers (MelanA, HMB45) and the myogenic marker desmin, leading to a diagnosis of malignant PEComa. Based on the presence of multiple worrisome features, the tumor was classified as malignant according to established criteria. Despite the absence of distant metastasis at initial imaging and surgery, the patient was offered adjuvant therapy but declined. She developed local recurrence and lung metastasis 72 days after surgery and died 169 days after surgery. This case highlights the clinical significance of a primary ovarian malignant PEComa and the importance of recognizing its potential for rapid and aggressive progression. The finding that multiple pathological risk factors predicted a poor clinical outcome reinforces the need for accurate diagnosis and prompt consideration of systemic therapy, such as mTOR inhibitors, in addition to surgical resection. This report contributes valuable insights into the clinical course of this rare tumor, underscoring the necessity of close follow-up and multidisciplinary management.

## Introduction

Perivascular epithelioid cell tumor (PEComa) is a rare mesenchymal neoplasm characterized by a distinctive histological feature [1]. Perivascular epithelioid cells show both smooth muscle and melanocytic differentiation [2]. While PEComas can arise in various gynecologic organs such as the uterus, ovary, and vulva, gynecologic PEComas account for approximately 25% of all reported cases [3,4], and primary malignant ovarian PEComas are extremely rare [5]. Due to the infrequent nature of this disease and lack of distinct symptoms, diagnostic procedures, as well as treatment options, are limited [6]. We report a case of a primary ovarian malignant PEComa with a poor prognosis that resulted in a rapidly progressive clinical course.

## Case presentation

A 73-year-old woman was referred to our department with a suspected left ovarian tumor, which was incidentally found during a CT scan for a fever and general malaise. The patient had a significant oncological history of two independent primary malignancies: left breast cancer at age 58 and lung cancer at age 68, both of which were treated with curative surgical resection. No adjuvant radiotherapy or systemic chemotherapy was administered for these prior cancers. There was no clinical or radiological evidence of recurrence of these tumors at the time of the PEComa diagnosis.

**Figure 1:**
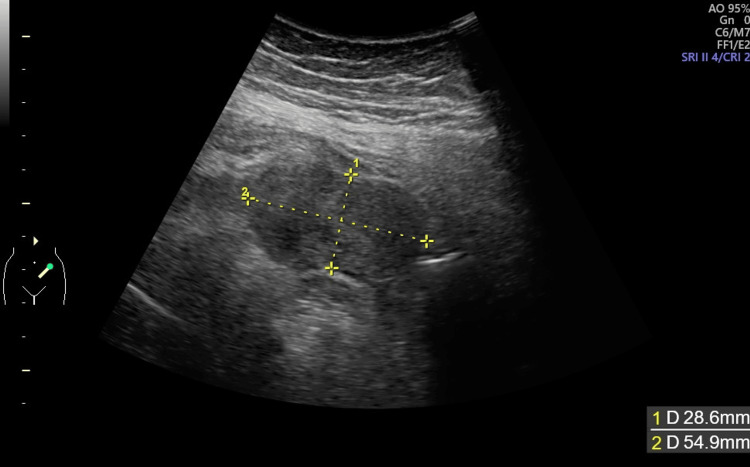
Transabdominal ultrasonography. A transabdominal ultrasound showing a 54 × 28 mm mass in the left lower abdomen.

Upon presentation, she had a temperature of 37.5°C. Abdominal palpation revealed no tenderness or palpable mass. A transabdominal ultrasound showed a 54 × 28 mm mass in the left lower abdomen (Figure 1), but a transvaginal ultrasound was unable to locate a mass. Laboratory tests showed an elevated C-reactive protein level of 11.98 mg/dL, while epithelial tumor markers (squamous cell carcinoma antigen, cancer antigen 125, carbohydrate antigen CA19-9, carcinoembryonic antigen) were within normal limits (Table 1).

**Table 1:** Blood investigation results. CRP: C-reactive protein; CEA: carcinoembryonic antigen; CA19-9: carbohydrate antigen 19-9; CA125: cancer antigen 125; SCC: squamous cell carcinoma antigen

Laboratory parameter	Patient value	Reference range	Units
CRP	11.98	0.00–0.14	mg/dL
CEA	<1.8	0.0–5.0	ng/mL
CA19-9	9	0–37	U/mL
CA125	8.1	0–35	U/mL
SCC	0.8	0.0–1.9	ng/mL

**Figure 2:**
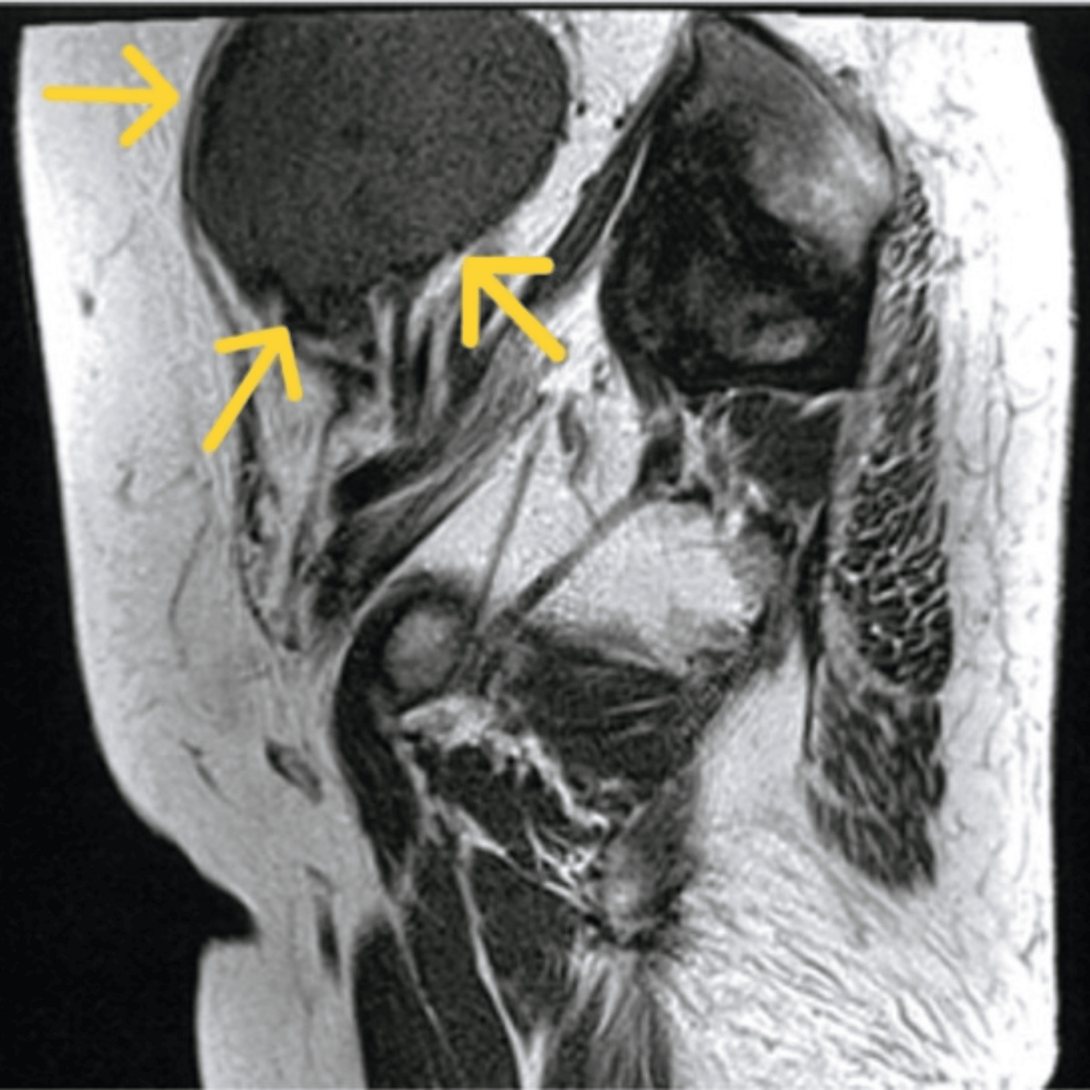
T2-weighted MRI scan. MRI demonstrating a 76 × 56 mm mass (yellow arrow) with slightly higher T2 signal intensity than the myometrium and low T1 signal intensity.

**Figure 3:**
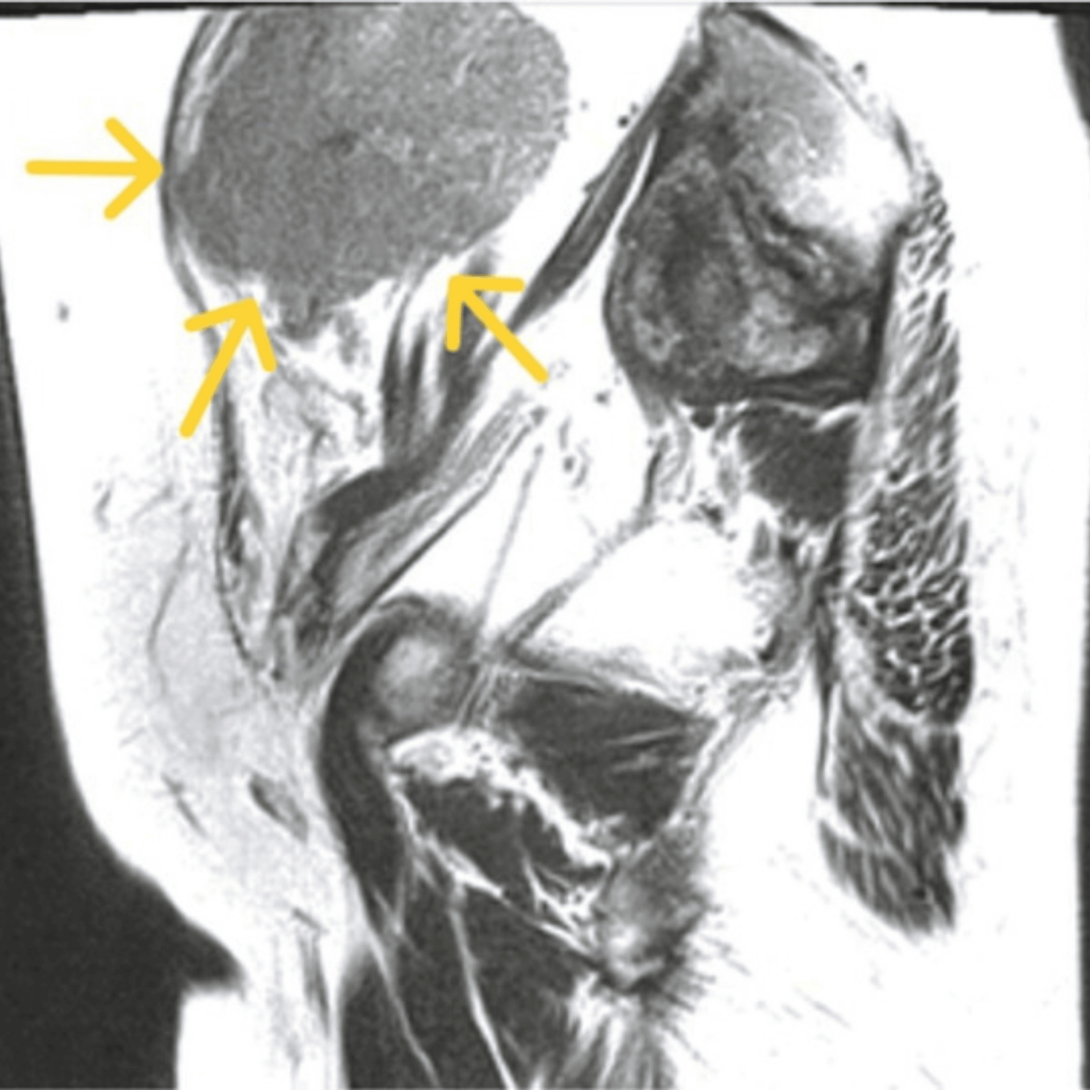
T1-weighted MRI scan. MRI demonstrating a 76 × 56 mm mass (yellow arrow) with slightly higher T2 signal intensity than the myometrium and low T1 signal intensity.

MRI revealed a 76 × 56 mm mass with a slightly hyperintense T2 signal and a hypointense T1 signal relative to the myometrium (Figures 2, 3).

**Figure 4:**
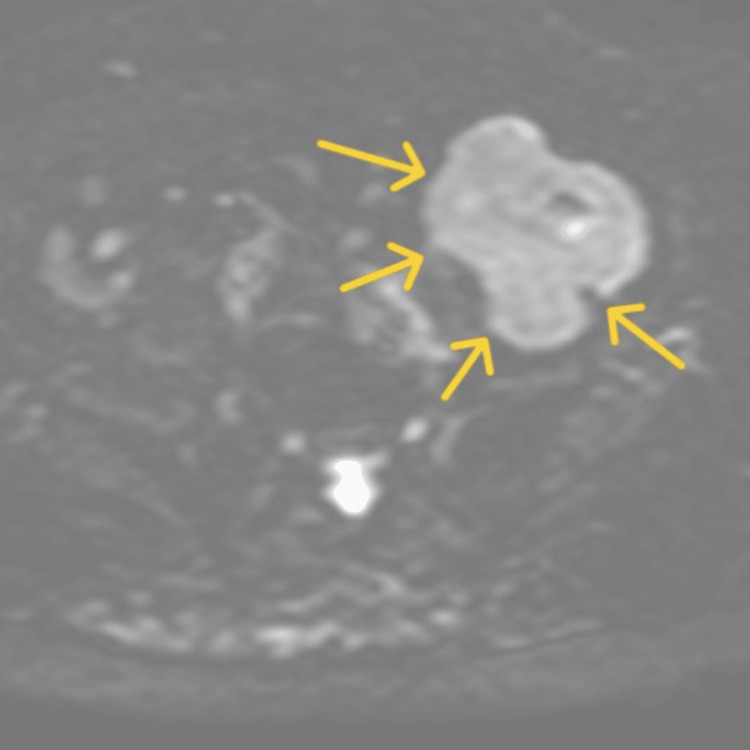
Diffusion-weighted MRI scan. The tumor (yellow arrow) showing high signal intensity on diffusion-weighted imaging, which was suggestive of malignancy.

In diffusion-weighted imaging, the tumor showed high signal intensity, supporting the suspicion of malignancy rather than a benign mesenchymal tumor (Figure 4).

**Figure 5:**
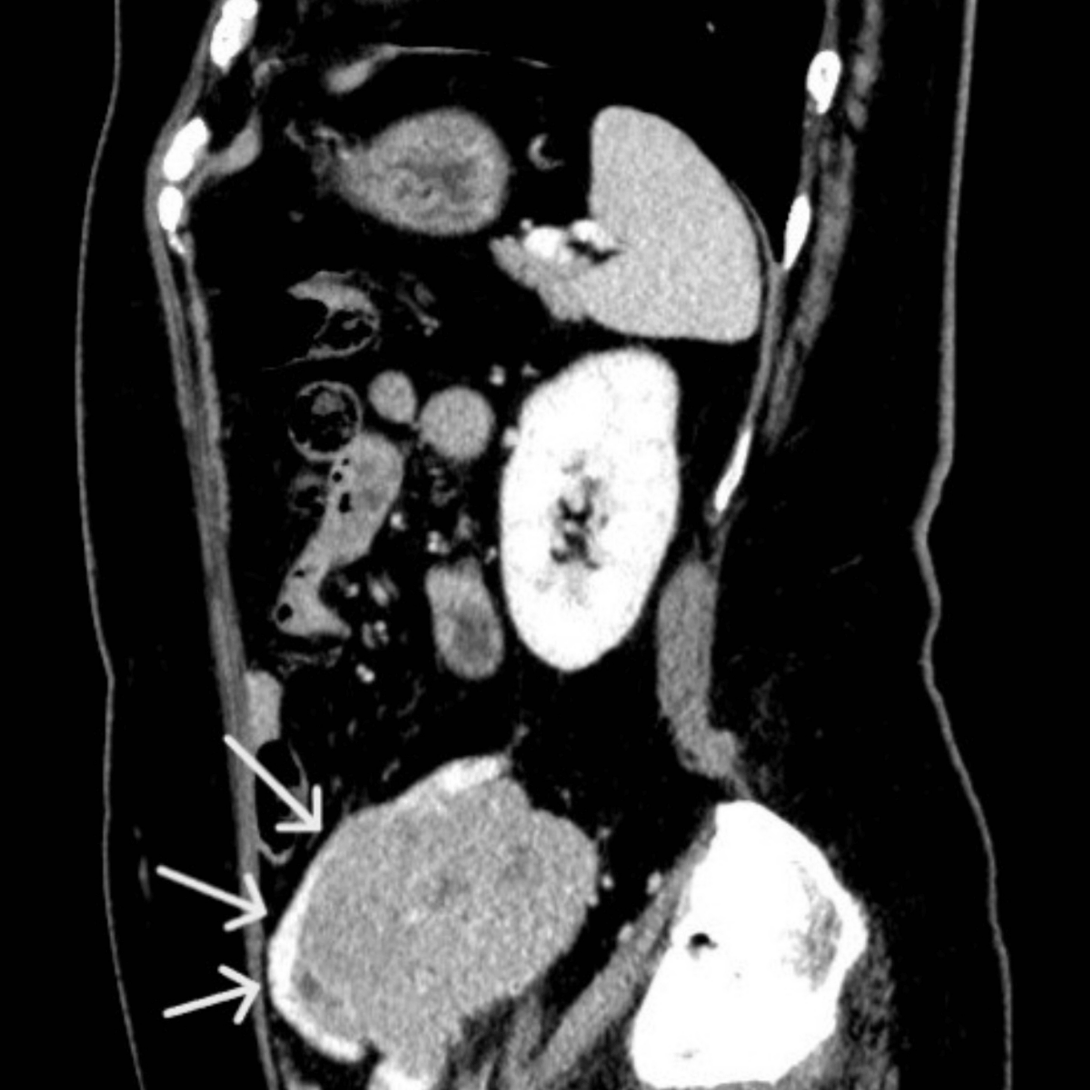
Contrast-enhanced CT (sagittal view). CT showing the left ovarian vein (white arrow) draining into the mass, suggesting an ovarian origin, with areas of poor enhancement indicating necrosis.

CT images indicated that the mass was a left ovarian tumor, as the left ovarian vein drained into it (Figure 5).

**Figure 6:**
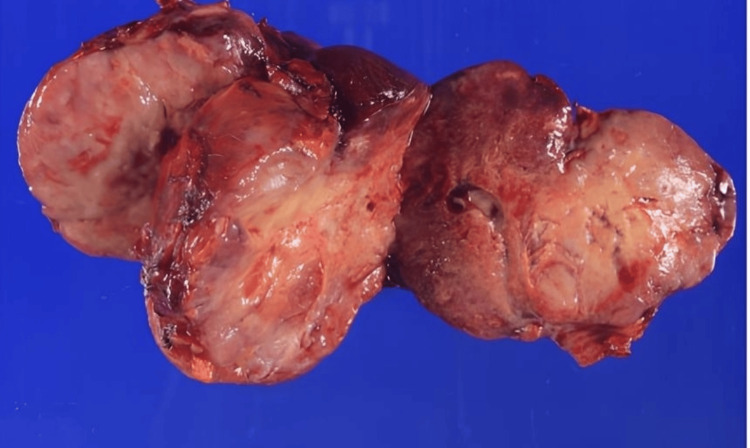
Macro image of the resected mass.

Based on the MRI and CT findings, a left ovarian malignant tumor was suspected, and the patient underwent surgery. The enlarged left adnexa was resected and sent for frozen section pathology, which was inconclusive for distinguishing between an epithelial and a non-epithelial tumor but suggested malignancy. Consequently, a total hysterectomy, completion of bilateral salpingo-oophorectomy (by removing the right adnexa), and partial omentectomy were performed. Peritoneal washings were performed and later confirmed to be negative for malignant cells. Although the intraoperative frozen section suggested malignancy, lymphadenectomy was not performed because the possibility of metastatic recurrence from the patient’s previous breast or lung cancers could not be entirely ruled out at that time. Given the diagnostic uncertainty regarding the primary site, we prioritized a less invasive approach. The resected tumor was a 9 × 7 × 7 cm solid mass with partial necrosis (Figure 6).

**Figure 7:**
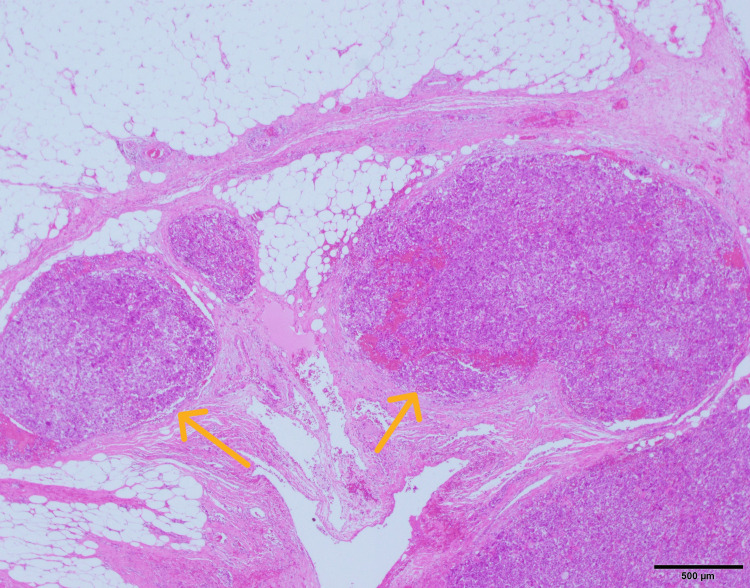
Hematoxylin and eosin-stained image (×40). Evidence of vascular invasion is present (yellow arrow).

**Figure 8:**
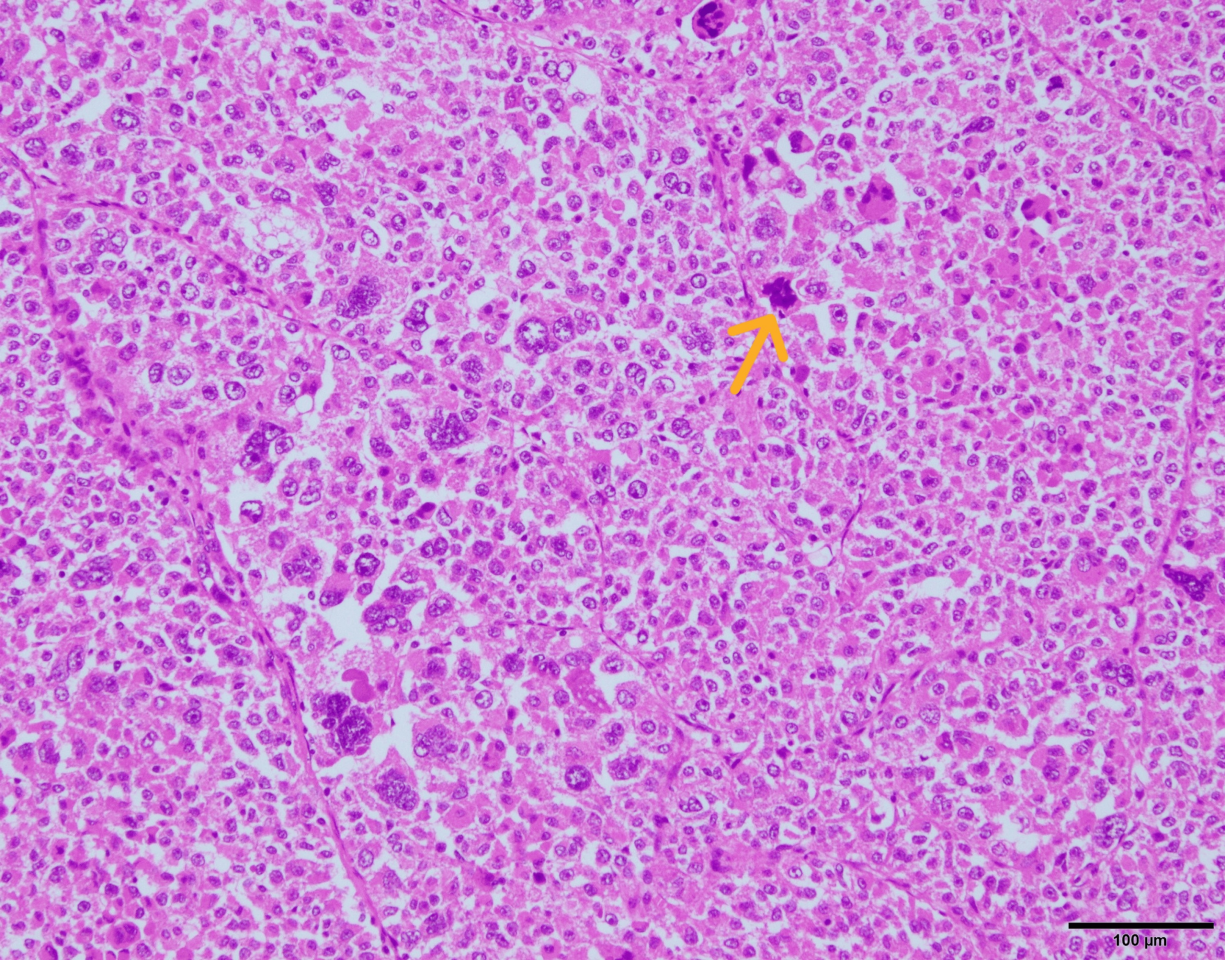
Hematoxylin and eosin-stained image (×100). Representative mitotic figures are shown (yellow arrow).

Histopathological examination revealed vascular invasion (Figure 7), a mitotic count of 10 mitoses per 10 high-power field (Figure 8), infiltrative growth, cytologic atypia, high mitotic rate, necrosis, and epithelioid cells with eosinophilic to clear cytoplasm.

**Figure 9:**
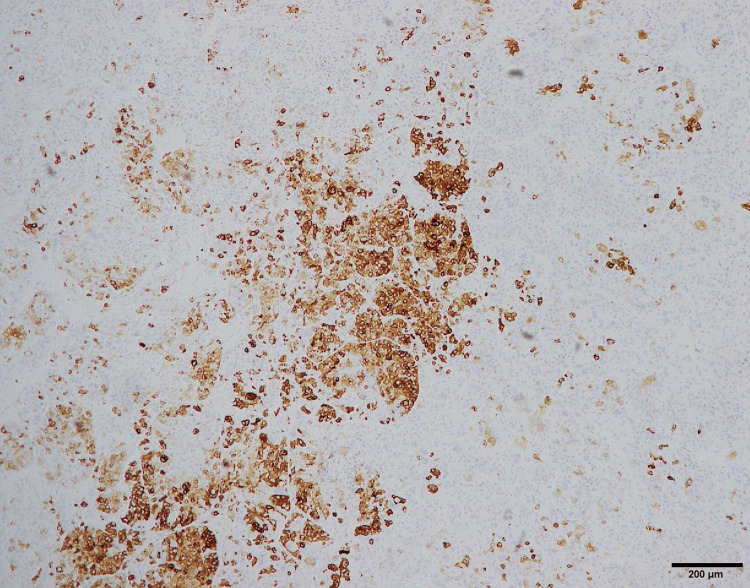
Immunohistochemical staining for MelanA.

**Figure 10:**
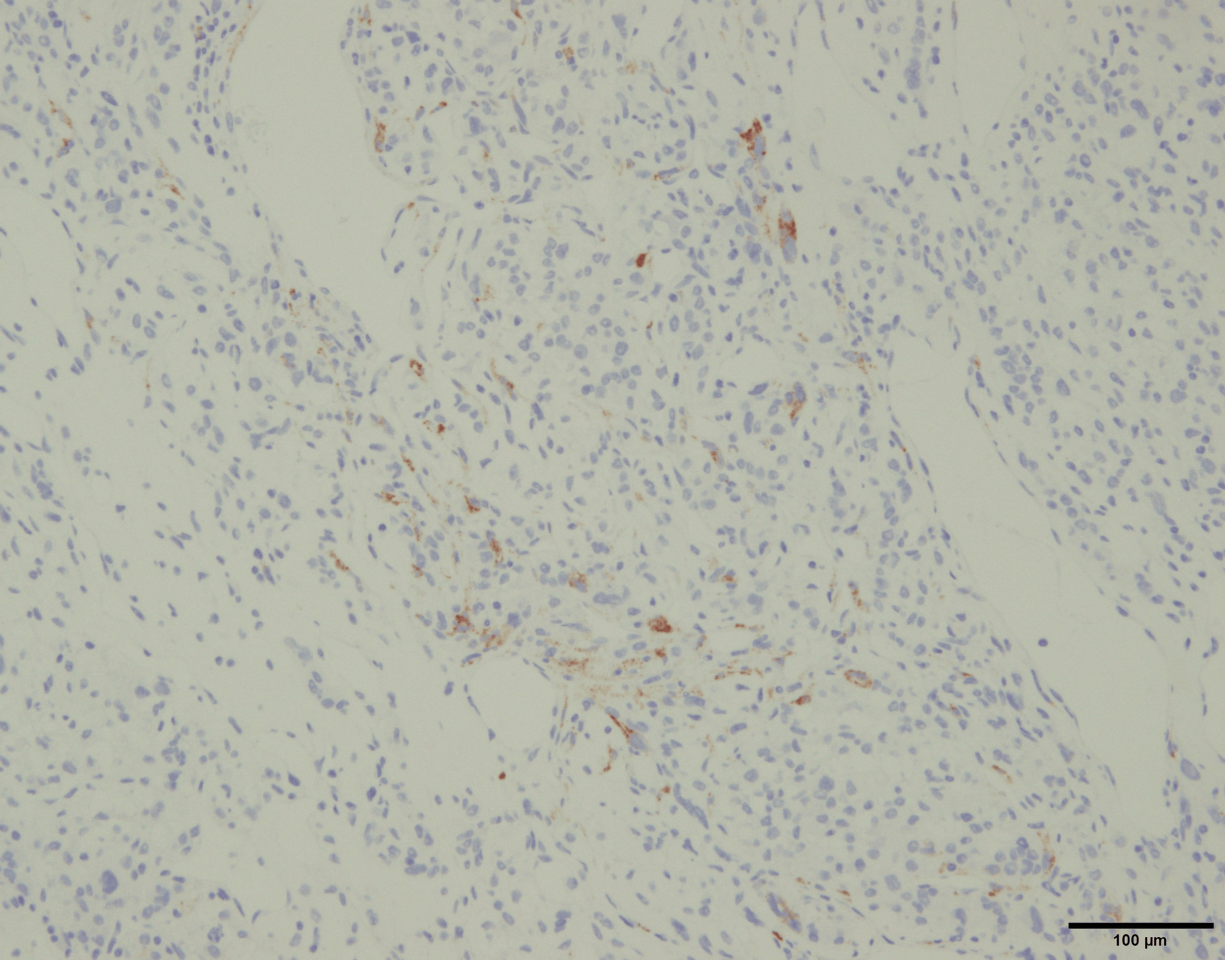
Immunohistochemical staining for HMB45.

**Figure 11:**
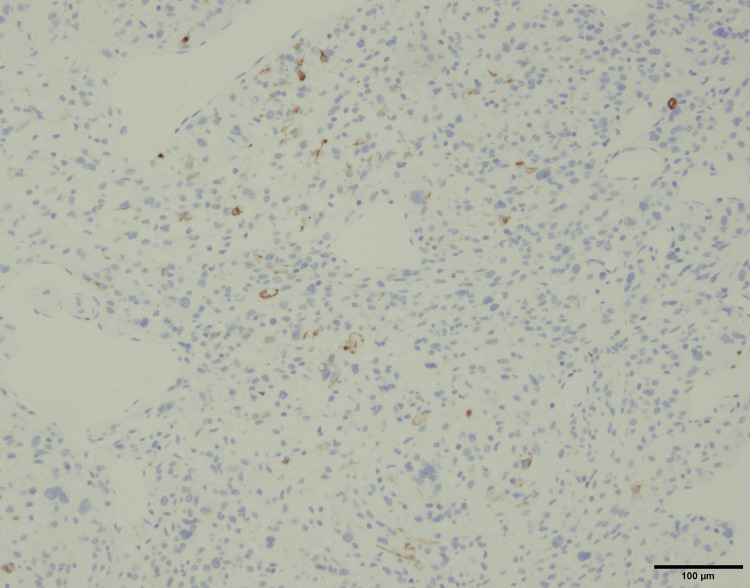
Immunohistochemical staining for desmin.

Immunohistochemical staining was positive for melanocytic markers MelanA and HMB45, as well as the myogenic marker desmin (Figure 9-11).

S-100 was negative. Based on the tumor size, infiltrative growth, cytologic atypia, high mitotic rate, necrosis, and vascular invasion, a diagnosis of malignant PEComa was made.

Postoperatively, we offered the patient carboplatin and paclitaxel chemotherapy, which was proposed based on standard ovarian carcinoma protocols, as no established regimen for malignant PEComa exists in Japan. However, the patient declined treatment. CT performed 72 days after surgery revealed a local recurrence on the left pelvic wall, measuring 5 × 3 cm, along with new lung metastases. Despite the aggressive disease progression, the patient’s condition deteriorated rapidly, and she passed away 169 days after surgery.

## Discussion

We report the case of a patient with a primary ovarian malignant PEComa with an extremely poor prognosis, who died approximately six months after surgery. Primary ovarian PEComa is exceedingly rare, and its clinical behavior is variable, with most reported cases being benign or borderline [7]. However, previously reported malignant cases have also shown rapid recurrence or death within a short period, consistent with our case [8].

The diagnosis of PEComa is often challenging, requiring differentiation from other tumors such as leiomyoma, epithelioid leiomyosarcoma, and melanoma [1,5-8]. Immunohistochemical analysis demonstrated positivity for MelanA and HMB45, both of which are melanocytic markers characteristic of PEComas. The tumor cells also showed focal positivity for desmin, indicating partial smooth muscle differentiation. The co-expression of melanocytic markers and smooth muscle markers represents a defining immunophenotypic feature of PEComa and is essential for establishing the diagnosis. This immunoprofile is particularly useful in differentiating PEComa from leiomyoma and epithelioid leiomyosarcoma, which typically lack melanocytic marker expression, as well as from malignant melanoma, which does not express smooth muscle markers such as desmin [9-11]. In our case, the diagnosis was based on the positive immunohistochemical staining for both myogenic and melanocytic markers, reflecting the tumor’s biological heterogeneity.

The assessment of malignancy in PEComas has evolved over time. Folpe et al. were the first to propose diagnostic criteria based on six worrisome features (tumor size >5 cm, infiltrative growth pattern, high nuclear grade and cellularity, mitotic rate ≥1 per 50 HPF, necrosis, and vascular invasion) [11]. Later, Schoolmeester et al. evaluated gynecologic PEComas and reported that tumors with four or more worrisome features could be considered malignant [12]. Subsequently, the Modified Folpe criteria were proposed, emphasizing that the presence of necrosis alone, or multiple risk factors, is sufficient for a malignant diagnosis [13]. Our case, which presented with multiple malignant factors, had a predicted poor prognosis. This pathological finding, consistent with the actual rapid clinical course, reinforces the importance of these factors in evaluating the malignancy of PEComas.

PEComas are characterized by the activation of the mTOR pathway due to mutations in *TSC1*/*TSC2* [14]. In recent years, mTOR inhibitors have garnered attention as a promising therapeutic strategy [15]. Clinical trials, such as the AMPECT study of nab-sirolimus, have demonstrated favorable results, including an objective response rate of 39%, a disease control rate of 71%, a median progression-free survival of 10.6 months, and a median overall survival of 40.8 months [16]. However, in Japan, mTOR inhibitors are currently not covered by national health insurance for the treatment of malignant PEComas. Due to these regulatory and financial constraints, we were unable to offer this molecularly targeted therapy to our patient. This case highlights the importance of including malignant PEComa in the differential diagnosis, especially in cases presenting with multiple risk factors. It also underscores the necessity of considering an aggressive treatment strategy from the outset. As this case illustrates, surgery alone may be insufficient, and the introduction of adjuvant therapy or molecularly targeted agents should be actively considered to improve the clinical outcome.

This report is limited by its single-case nature, which restricts the ability to generalize the findings. In addition, molecular analyses were not performed, and standardized treatment strategies for malignant PEComa remain undefined. Nevertheless, this case provides important clinical insight into the potential for rapid progression in primary ovarian PEComa.

## Conclusions

Primary ovarian PEComa is a rare mesenchymal tumor with variable clinical behavior. This case highlights that, although many reported cases show indolent features, aggressive progression with early recurrence and metastasis may occur. Accurate diagnosis relies on appropriate histopathological and immunohistochemical evaluation. Further accumulation of cases is necessary to better understand the biological behavior and establish optimal management strategies for this rare entity.
